# Occurrence of *Helicobacter pullorum* in Retail Chicken Meat: A One-Health Approach to Consumer Health Protection

**DOI:** 10.3390/foods13060845

**Published:** 2024-03-10

**Authors:** Nicoletta C. Quaglia, Flavia Capuozzo, Federica Ioanna, Michele De Rosa, Angela Dambrosio

**Affiliations:** Department of Veterinary Medicine, University of Bari “Aldo Moro”, Strada Prov.le per Casamassima, Km 3, 70010 Valenzano, Bari, Italym.derosa1986@gmail.com (M.D.R.);

**Keywords:** *Helicobacter pullorum*, retail chicken meat, poultry, foodborne pathogens, PCR, Good Hygiene Practices, food safety

## Abstract

*Helicobacter pullorum* is an emerging foodborne pathogen that commonly colonizes the gastrointestinal tract of poultry, causing gastroenteritis. It has been related to several clinically important infections, including colitis and hepatitis, inflammatory bowel disease, recurrent diarrhea, and bacteremia in the human population. The bacterium may be transmitted to humans through undercooked poultry meat. In order to investigate the occurrence of *H. pullorum* in raw retail chicken meat (thighs and breasts), we analyzed 240 samples: 120 chicken thigh and 120 chicken breast samples. The samples were analyzed by means of an isolation protocol using Steele and McDermott’s modified filtration technique on Brucella agar supplemented with 5% of defibrinated sheep’s blood. The presumptive colonies were biochemically identified and analyzed using a previously described conventional PCR test based on the 16S rRNA gene. In total, 35% of analyzed samples were positive using the microbiological protocol and 45% were positive by PCR. These results suggest that *H. pullorum* can be transmitted to humans through the handling and consumption of raw poultry meat, representing a risk for food business operators and consumers. Efforts to control *H. pullorum* in broiler meat should prioritize the implementation of stringent hygienic practices across all stages of the food chain, from the farm to the consumer.

## 1. Introduction

Over the past two decades, the poultry industry has expanded globally due to increased consumer demand for white meat [1]. For this reason, poultry has become the most widely consumed source of meat, and this has increased the number of foodborne and zoonotic diseases, sometimes life-threatening, transmitted through this type of meat, which is a significant health problem worldwide [1,2,3]. The consumption of undercooked and poorly processed meat is the source of foodborne diseases, especially in poultry meat. Contamination of meat often occurs during rearing, poultry slaughter, and handling during cutting or processing [4]. Among the foodborne pathogens typical of poultry meat, such as *Salmonella enterica*, *Campylobacter* spp., and Shiga toxin-producing *Escherichia coli*, *Helicobacter pullorum* (*H. pullorum*) has been identified as an emerging infectious agent. It was first isolated from the feces of poultry and humans by Stanley et al. [5]. *H. pullorum* belongs to the genus *Helicobacter* and it is a fastidious, microaerophilic, non-sporulated, and Gram-negative spirally curved motile bacillus with monopolar flagellae [6]. It is bile-resistant and requires a microaerobic environment supplemented with H_2_ in which growth occurs at 37 and 42 °C [5]. It belongs to the enterohepatic *Helicobacter* species (EHH) that colonize the host intestine and hepatobiliary system [7]. *H. pullorum* naturally infects many poultry birds, some rodent species, and also humans. It has been isolated from the liver, duodenum, and appendix of asymptomatic poultry [3,5,8,9] and has been associated with enteritis and vibrionic hepatitis in broilers and laying hens [10]. Furthermore, it has been isolated from the feces of human patients with enteritis as well as healthy humans [11,12].

*H. pullorum* is associated with several clinically significant infections in humans [13], such as colitis and hepatitis, inflammatory bowel disease, recurrent diarrhea, and bacteremia [14,15]. *H. pullorum* is also thought to play an important role in the development of Crohn’s disease [16].

Therefore, although it represents a public health problem, to date, evidence on the presence of *H. pullorum* in chicken meat is poor and incomplete [17]. However, the isolation of *H. pullorum* at high levels in the cecum of chickens, its excretion in droppings till slaughter [11] and consequently of its occurrence on poultry carcasses, which are likely contaminated during slaughter [17], implies that chicken meat is most likely the main source of infection for humans [11]. All this evidence makes it one of the most important emerging foodborne pathogens [4].

The major reservoirs of this bacterium are commercially reared poultry, especially broilers, turkeys, laying hens, and quail [10,18,19,20]. In particular, the prevalence of *H. pullorum* has been found to be relatively high in poultry farms, and the prevalence rate in poultry (especially in slaughter-age broiler flocks) can be as high as 100% in ceca, 47% in liver, and 23.5% in meat samples [9,17,21].

Therefore, one of the most likely routes of *H. pullorum* transmission to humans is the cross-contamination of food at different stages of the food chain, e.g., during slaughter, which may contaminate the carcass with intestinal contents. In addition, contamination of chicken meat can also occur during transport, food handling, preparation, and even consumption [9,19].

Studies on the occurrence of the bacterium in retail chicken meat samples are still limited and are mainly focused on Iran, less so on other countries. *H. pullorum* has been isolated in some chicken meat products, including breasts, thighs, livers, and wings [10,17,18,22,23,24,25].

The reasons for the lack of data on the presence of the bacterium in poultry meat products may be that there is no standardized method for its isolation in contaminated food and that the identification of the bacterium is difficult and time-consuming.

Recovery of *H. pullorum* can be optimized if samples are as fresh as possible and non-selective methods or selective media without polymyxin B are used [9]. Moreover, the best strategy to identify this organism, as well as all bacteria of the genus *Helicobacter,* seems to be the use of a combination of phenotypic and genotypic methods [26].

Due to the lack of studies on the presence of *H. pullorum* in retail chicken meat, the aim of this study was to evaluate the presence of the pathogen in samples of raw retail chicken thighs and breasts to assess the risk to the food business operator and consumer from both cross-contamination and consumption of undercooked meat. The samples were analyzed by means of an isolation protocol using the modified filtration technique of Steele and McDermott [27] and a previously described conventional PCR assay based on the 16S rRNA gene [5] for the isolation and detection of *H. pullorum*.

## 2. Materials and Methods

### 2.1. Sampling

A total of 240 samples of raw chicken meat (120 chicken thighs and 120 chicken breasts samples) were randomly collected from various retail markets and supermarkets in southern Italy (Apulia region) and analyzed from September 2021 to April 2023. At the time of purchase, all samples of chicken thighs and breasts was packaged in polystyrene containers sealed with plastic film.

Each sample was aseptically collected and delivered to the laboratories in a refrigerated box at ca. 4 °C and immediately analyzed. All samples were subjected to microbiological analysis and PCR assay based on the 16S rRNA gene [5] for isolation and detection of *H. pullorum*.

### 2.2. Bacteriological Isolation

From each chicken thigh or breast sample, 25 g of raw meat was aseptically collected and added to 225 mL of brain heart infusion broth (Liofilchem, Teramo, Italy) supplemented with 10% of sterile inactivated horse serum and vancomycin (10 mg/L), trimethoprim (5 mg/L) and amphotericin B (5 mg/L) (Sigma Aldrich, Milano, Italy). Before use, the serum was inactivated at 56 °C for 30’ in order to inactivate the complement system, which could interfere with bacterial growth. Horse serum is a protein-rich blood derivative that allows for the optimization of bacterial growth, particularly when low bacterial loads are present [28].

The samples were homogenized with the enrichment broth in a stomacher for 2 min, then incubated in microaerobic conditions (10% CO_2_, 5% O_2_ and 85% N_2_) in a 3.5 L anaerobic system jar with an Oxoid™ CampyGen™ 3.5 L Sachet (Thermo Fisher, Monza, Italy) at 37 ± 1 °C for 24–48 h.

After 48 h, the samples were inoculated in duplicate on Brucella agar (Liofilchem) supplemented with 5% defibrinated sheep’s blood (Liofilchem) using the modified filter technique of Steele and McDermott [27]. In brief, 300 µL of each broth culture was spread on a 0.45 µm pore size, 47 mm diameter cellulose nitrate membrane filter (Axiva Sichem Biotech, Dehli, India) previously placed on the Brucella agar surface. The plates were incubated at 41.5 ± 1 °C for 1 h in microaerobic conditions in a 2.5 L anaerobic system jar with an Oxoid™ CampyGen™ 2.5 L Sachet (Thermo Fisher, Italy). After the incubation period, the filter was gently removed and the inoculum was streaked with a loop on the Brucella agar surface. The plates were then incubated in a microaerobic atmosphere in an anaerobic system jar as described above at 41.5 ± 1 °C for 7 days and examined daily for growth.

### 2.3. Biochemical Identification

Five colonies with *H. pullorum*-like appearance, i.e., small greyish-white colonies, were chosen and harvested from the samples that showed bacterial growth. The colonies were subcultured on Brucella agar supplemented with 5% defibrinated sheep’s blood and incubated at 41.5 ± 1 °C for 72 h in microaerobic atmosphere in an anaerobic system jar, as described above, and subjected to biochemical identification. Presumptive recognition of the bacterium was performed by Gram staining, observation under an optical microscope (S-shaped curved rod), catalase and oxidase reactions, nitrate reduction, urease test, growth in the presence of 2% NaCl, and sensitivity to nalidixic acid [19,25,29]. The latter two tests were used to discriminate *H. pullorum* from *Campylobacter lari*. All colonies presumptively identified as *H. pullorum* were subjected to a PCR test based on the 16S rRNA gene [5] for definitive identification.

### 2.4. PCR Analysis

PCR was performed on all broth cultures and on all colonies presumptively identified as *H. pullorum*.

Bacterial DNA was extracted from 1 mL of broth culture from each sample after 48 h of incubation, and from the colonies presumptively identified as *H. pullorum* using a GeElute^™^ Bacterial Genomic DNA kit (Sigma-Aldrich, Milano, Italy) according to the manufacturer’s instructions.

In order to detect the 16S rRNA gene of *H. pullorum*, specific primers (forward, 5’ ATG AAT GCT AGT TGT TGT CAG 3’; reverse, 5’ GAT TGG CTC CAC TTC ACA 3’) (TIB Molbiol S.r.l., Genoa, Italy) targeting the 447 bp fragment were employed [5]. Samples (2 µL) of each extract were amplified in 25 µL GoTaq^®^ Green Master Mix (Promega Corporation, Madison, WI, USA) and 0.5 µL of each primer. PCR amplification was performed according to the following protocol: 95 °C for 2 min and 35 cycles at 94 °C for 1 min, 60 °C for 2 min, 72 °C for 1.5 min, followed by 72 °C for 5 min. In the current study, the PCR-amplified products (10 μL) were subjected to electrophoresis in 1.5% agarose (Sigma-Aldrich, TaufKirchen, Germany) gel with 100 bp Plus DNA Ladder (Fermentas, Leon-Rot Germany) for amplicon determination.

### 2.5. DNA Sequencing

In order to achieve the confident identification of the amplicons, all PCR products were purified using Ultrafree-DA columns (Amicon, Millipore, Milan, Italy) and sequenced in ABI-PRISM 377 (AEM Bioscience, London, UK) using the Taq Dye Deoxy Terminator Cycle Sequencing Kit (Applied Biosystems, Foster City, CA, USA). Sequences were determined in both directions using the same primers as for the PCR. The resulting nucleotide sequence of 447 bp of the 16S rRNA gene was aligned using the Basic Local Alignment Search Tool (BLAST, National Center of Biotechnology Information—NCBI) and compared with known sequences previously deposited in a publicly available database on the NCBI server.

### 2.6. Statistical Analysis

The data were analyzed using Microsoft Excel software 16.16.27 201012 (Windows, 2010). A two-way analysis of variance (ANOVA) was performed to evaluate the differences between the number of positive thigh samples and the number of positive breast samples and between the number of samples positive by bacteriological analysis and the number of samples positive by PCR, with statistical significance set at *p* < 0.05.

## 3. Results

The results are summarized in Table 1.

Out of 240 raw chicken meat samples analyzed by the culture method, 84 samples (35%) were positive for the presence of *H. pullorum*. Specifically, 51 positive samples were chicken thighs (42.5% of the 120 samples analyzed) and 33 positive samples (27.5% of the 120 samples analyzed) were chicken breasts.

Of the 420 colonies selected from the 84 positive samples (255 from the 51 positive chicken thigh samples and 165 from the 33 positive chicken breast samples), 350 (83.3%) were presumptively identified as *H. pullorum* by phenotypic and biochemical tests (216 of the 255 colonies from the positive chicken thigh samples (84.7%) and 135 of the 165 colonies from the positive chicken breast samples (81.8%)). The selected colonies had the following phenotypic and biochemical characteristics: punctate, non-pigmented, translucent, and alpha-hemolytic; Gram-negative; positive for oxidase, catalase, and nitrate reduction; negative for urease production; intolerant to 2% NaCl; and sensitive to nalidixic acid.

**Figure 1:**
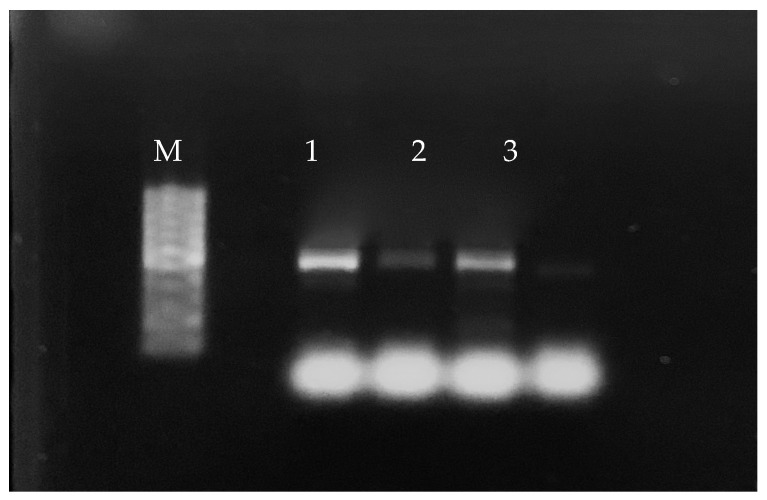
Results of the PCR analysis of some broth culture enrichment samples of commercial chicken thighs and breasts. Legend: M: 100 bp Plus DNA Ladder; 1 and 2: chicken thigh samples; 3: chicken breast sample.

All 350 colonies presumptively identified as *H. pullorum* produced the expected 447 bp amplicon in the PCR assay based on the 16S rRNA gene (Figure 1).

Of the 240 enrichment broth cultures incubated for 48 h, 108 (45%) were positive for *H. pullorum* by PCR. Specifically, 66 positive enrichment broth cultures (55%) were from the 120 chicken thigh samples and 42 (35%) were from the 120 chicken breast samples (Figure 1). All samples positive by the bacterial isolation protocol (84 samples in total) were also positive by PCR.

PCR amplicon sequences showed 97–99% identity with the 16SrRNA gene sequence of *H. pullorum*.

Statistical analysis revealed no significant differences between the results of chicken thigh and breast analysis (*p* > 0.05) and between the results obtained by the two methods of analysis (*p* > 0.05).

## 4. Discussion

*H. pullorum* has recently emerged as a foodborne pathogen of public health concern [17]. It has been isolated in high concentrations from the cecal contents of healthy broilers, from the liver and intestinal contents of laying hens with vibriohepatitis, from the feces of human patients with enteritis, and from clinically healthy humans [9,19,20]. The reservoirs and routes of transmission to humans are not yet well established, but the most widely accepted hypothesis is cross-contamination of food throughout the food chain, beginning with slaughter, meat transport, handling, and ending with processing and consumption [9,19].

*H. pullorum* was discovered in the early 1990s by Stanley et al. [5], but data on the prevalence of the pathogen in retail chicken meat in different regions of the world remain limited and inconsistent [17]. As far as we know, there are few investigations on the presence of *H. pullorum* in commercial chicken meat samples, and most of them were conducted in Iran. For example, Jebelli et al. [25] analyzed 50 samples of chicken thighs by PCR and by the culture method and obtained 16% positive results. In another study, the authors analyzed 60 chicken wing samples and 18% of these tested positive [23], while 40% of raw chicken meat samples (mainly chicken thighs and breasts) tested positive in another recent study [22]. In the latter two studies, the culture method was combined with PCR. The inconsistent and limited data on the presence of *H. pullorum* in retail chicken meat could be due to misclassification as other enteric agents, particularly thermophilic *Campylobacter* species, due to their similar phenotypic and genetic characteristics and lack of standardized protocols [1].

Therefore, evaluating the presence of the bacterium in retail chicken meat is important for careful risk assessment to reduce contamination. As of now, much of the research has focused on the occurrence of the bacterium in cecal contents as the primary site of *H. pullorum* colonization in poultry.

However, research in this area should also focus on the risk to consumers of contracting the bacterium through consumption of contaminated poultry meat and the preventive measures that should be taken from primary production to the consumer’s table.

To this purpose, we processed 240 retail samples of raw chicken meat: 120 chicken thigh and 120 chicken breast samples. A total of 35% of the samples were positive using the isolation protocol described above, while the detection rate was 45% when broth cultures were analyzed by PCR. The isolation rates are consistent with those of other authors, who isolated *H. pullorum* in 0% to 49% of samples [4]. However, the data are not always easy to compare due to the differences between the samples analyzed (fresh or frozen), the different analytical methods, and the limited number of investigations conducted so far.

In particular, it is known that the chance of isolating the bacterium from food increases when fresh samples are analyzed [30]. This is likely due to the possibility that *H. pullorum*, like other bacteria belonging to the genus *Helicobacter*, e.g., *H. pylori*, is able to overcome the stress conditions that occur outside and inside the host and transition to the viable but non-culturable (VBNC) state. When this morphological change occurs, the bacterium is unable to grow on solid medium using conventional culture methods. This can lead to false negative results, underestimating the presence of the bacterium in food [26]. Further research is needed in this field in order to evaluate the conversion to VBNC and its role in the transmission of the pathogen to humans through the consumption of contaminated food.

Another potential reason could be the low survival ability of the bacterium on food, but there are no data confirming this hypothesis.

In addition, to date, there is no standardized protocol for the selective culture of *H. pullorum* due to its “fastidious nature”, with special growth requirements and a long incubation period, as well as correct sample transport at refrigeration temperature [19]. Several isolation protocols have been described; they involve several culture media, two different incubation temperatures (37 or 42 °C), and various selective supplements [9,17,19,22]. Furthermore, the biochemical identification of *H. pullorum* is difficult as it is a microorganism biochemically similar to other bacteria belonging to the *Campylobacter* genus [31].

However, for better recovery of *H. pullorum* from food, the use of non-selective methods such as the modified filter technique of Steele and McDermott or selective media without polymyxin B (to which *H. pullorum* is sensitive) has been recommended [32].

Therefore, our high isolation rates (35% with the culture method and 45% with PCR), could be due both to the storage of the samples at 4 °C and their analysis immediately after delivery to the laboratories, and to the selective isolation protocol used. The latter included a selective enrichment step to reduce the contaminating bacterial load, followed by PCR assay after the incubation period. Moreover, the isolation protocol involved the use of a filter with a 0.45 µm pore size, and not 0.65 µm, as suggested by other authors [17], which may have lowered the passage of contaminating bacteria. In addition, incubation in microaerobic conditions for 1 h at 41.5 °C before the removal of the filter may have increased the motility and viability of the microorganism [9].

Furthermore, it has been proposed that the best strategy for identifying the microorganism is a combination of a conventional culture method and PCR assay [29]. In fact, biochemical tests allow for the presumptive identification of the bacterium as it is biochemically and phenotypically very similar to some *Campylobacter* species. In particular, it is biochemically close to *Campylobacter lari*, except for intolerance to 2% NaCl and sensitivity to nalidixic acid [31]. These two tests are important as they allow for the screening of isolated bacteria on which PCR can subsequently be performed in order to obtain rapid confirmation of the identity of the bacteria isolated. To confirm this, the biochemical identification tests we performed proved to be sensitive and specific as all colonies selected with presumptive identification of *H. pullorum* were confirmed by PCR. It is therefore essential to add the tests for intolerance to 2% NaCl and sensitivity to nalidixic acid to the isolation protocol. According to The European Union One Health 2021 Zoonoses Report by the EFSA and the European Centre for Disease Prevention and Control, campylobacteriosis is the most commonly reported zoonosis in Europe, followed by salmonellosis [33]. In addition, data from slaughterhouse surveillance of broiler carcasses reported a positivity of 31%, with 18.4% of samples exceeding the limit of 1000 CFU/g [33]. It is likely that due to the difficulty in isolating *H. pullorum* and its biochemical proximity to microorganisms of the *Campylobacter* genus, the presence of *Campylobacter* spp. is overestimated because it is not easily distinguished from *H. pullorum*. In view of what has been said so far, systematic monitoring of the presence of *H. pullorum* in slaughterhouses would be desirable in order to also perform a more accurate risk assessment for the presence of *Campylobacter* spp. in chicken meat. The development of a specific and sensitive isolation protocols is therefore of paramount importance.

PCR, being a more sensitive and rapid method than microbiological analysis alone, has become increasingly important for food analysis for public health purposes. Indeed, the rapid identification of foodborne pathogens plays an important role in the early detection and treatment process of these pathogens. Although culture-based methods are still the gold standard in food analysis, molecular methods aid in the diagnosis and molecular epidemiology of foodborne pathogens [19]. The benefits of molecular tests are high sensitivity and specificity, reduced final costs of detection tests, and rapidity of execution, especially when fastidious pathogens are concerned [2,9,19]. Furthermore, in the case of *H. pullorum,* for which identification is uncertain, PCR helps to avoid the over- or underestimation of the presence of the bacterium in foods. It is true that the 24 additional positive samples compared to the culture method (84 positive samples with the culture method versus 108 positive samples by PCR) could only be due to the presence of DNA from bacteria that are no longer alive and viable, but it could also be due to the presence of VBNC *H. pullorum*.

Therefore, is important to use PCR in the protocol both for a confident identification of isolates and for obtaining epidemiological data. However, the selective isolation protocol used in this survey was also specific, as all 28 positive samples were confirmed by PCR.

Statistical analysis showed no significant difference between the results obtained with the two analytical methods. This finding confirms that the analysis of fresh samples and the protocol applied were important in avoiding pathogen death and conversion to VBNC. Although the result is not statistically significant, it should be emphasized that PCR allowed for the identification of 24 positive samples that had been considered negative on bacteriological analysis alone.

The statistical analysis of the positive results between the thigh (42.5% and 55% with the microbiological and PCR protocol, respectively) and breast samples (27.5% and 35% with the microbiological and PCR protocol, respectively) also gave no significant difference. The reason could be cross-contamination within the slaughterhouse, and cutting and packing may have further contributed to the spread of contamination throughout the carcass.

There are several studies on the presence of *H. pullorum* in the ceca contents of apparently healthy broilers reporting isolation rates from 4% to 100% depending on geographical region and farming practices [2,6,9,20], but there are no data correlating the presence of the pathogen in the cecum and on the broiler carcass. However, the high isolation rates from the gastrointestinal tract of chickens have led several authors to assume that *H. pullorum* is able to contaminate carcasses during slaughter and this implies that chicken meat constitutes a major source of infection for humans [30,34]. Our results show that the bacterium is present on retail chicken meat, probably as a result of contamination of carcasses and subsequently by cross-contamination during processing and handling.

As is recognized for *Campylobacter* spp., in order to reduce the risk for the consumer, control measures for *H. pullorum* should be taken during primary production in order to reduce human exposure by decreasing the contamination of chicken meat along the food chain [34]. Any control strategy for *H. pullorum* in chicken meat should be based on the application of Good Hygiene Practices (GHPs) at all stages of the food chain and monitoring their effectiveness in preventing meat contamination, as has been widely recommended for the prevention of *Campylobacter* spp. transmission to humans [34,35]. Furthermore, control of the pathogen on broiler farms may have a great impact on public health. Based on these considerations, it is important to prevent the entry of *H. pullorum* into broiler flocks during primary production, as has been suggested for *Campylobacter* spp. [36]. The approach that should be taken is based on biosecurity, on increasing the resistance of broilers to colonization by adding organic acid additives and phytocompounds to drinking water and/or feed, and on reducing the concentration of *H. pullorum* in the intestines of broilers before slaughter, e.g., by treatment with bacteriophages or bacteriocins. However, more information is needed about the effectiveness of this approach for poultry farms, and about the impact of these measures on human health [34].

Improved hygienic measures are also essential during the transport of live birds and during the slaughtering and dressing of carcasses, where contamination of carcasses may occur through fecal matter spillage or cross-contamination.

Finally, it is important to inform food business operators about hygienic practices in professional and domestic catering, with the main objective of preventing cross-contamination when handling chicken meat.

Further research is needed to assess whether these control measures are effective in preventing the contamination of meat by *H. pullorum*.

## 5. Conclusions

*H. pullorum* colonizes the cecum of chickens in high concentrations and has been isolated on chicken carcasses, probably due to contamination during slaughter. Therefore, the potential role of this bacterium as an emerging foodborne pathogen must be considered. However, data on the presence of *H. pullorum* in commercial chicken meat are poor and inconsistent due to the lack of standardized protocols. The PCR-confirmed isolation of the bacterium in 45% of analyzed retail meat samples suggests that *H. pullorum* could be transmitted to humans through contact with or consumption of undercooked chicken meat.

These results emphasize the importance of focusing research on this emerging foodborne pathogen. In addition, the presence of *H. pullorum* on chicken carcasses may be a cause of overestimating the presence of *Campylobacter* spp., which, despite monitoring at the slaughterhouse, is the most common foodborne disease reported in Europe. Therefore, control of *H. pullorum* and *Campylobacter* spp. throughout the food chain is of paramount importance.

Then, it is essential to focus on methods to control this pathogen from farm to retail, also considering the importance of providing consumers with information on the application of Good Hygiene Practices when handling raw chicken meat in order to avoid cross-contamination. These data will be of public health significance in relation to reducing human exposure associated with handling and consumption of contaminated processed chicken meat. Further research is needed to evaluate the survivability of the pathogen in chicken meat and other foods, including ready-to-eat foods that may be contaminated by cross-contamination. In addition, it is important to develop specific and sensitive standardized isolation protocols able to recover the pathogen from complex foodstuffs.

## Figures and Tables

**Table 1:** Samples of raw chicken meat and results with microbiological and PCR assays.

Samples	*n*. of Analyzed Samples	Positive Samples *n*. (%)	Broth Culture Positive to PCR *n*. (%)	Colonies Biochemically Identified as *H. pullorum n*. (%)
chicken thighs	120	51 (42.5%)	66 (55%)	216 (84.7%) *
chicken breasts	120	33 (27.5%)	42 (35%)	135 (81.8%) *
	240	84 (35%)	108 (45%)	350 (83.3%)

* The percentage is calculated for 255 colonies selected from the 51 positive thigh samples and 165 colonies selected from the 33 positive breast samples.

## Data Availability

The original contributions presented in the study are included in the article, further inquiries can be directed to the corresponding author.
